# Porosity induced by dislocation dynamics in quartz-rich shear bands of granitic rocks

**DOI:** 10.1038/s41598-022-10053-x

**Published:** 2022-04-12

**Authors:** Jacques Précigout, Estelle Ledoux, Laurent Arbaret, Charlotte Spriet

**Affiliations:** 1 Institut des Sciences de la Terre d’Orléans (ISTO), Université d’Orléans, CNRS, BRGM, UMR7327, 45071 Orléans, France; 2 Unité Matériaux et Transformation (UMET), Université de Lille, CNRS, UMR8207, 59655 Villeneuve d’Ascq, France; 3 Institut de Physique du Globe de Paris (IPGP), Université de Paris, CNRS, UMR7154, 75238 Paris, France

**Keywords:** Structural geology, Solid Earth sciences, Tectonics

## Abstract

The production of micro-pores is a driving mechanism for fluids to interact with deep environment and influence rock properties. Yet, such a porosity still remains misunderstood to occur in viscous rocks and may be attributed to either grain boundary sliding (GBS), dissolution effects or sub-grain rotation. Here we focus on quartz-rich shear bands across the Naxos western granite (Aegean Sea, Greece), where we document sub-micron pores at quartz boundaries. While most of these pores are observed along grain boundaries, some of them occur at intra-grain boundaries, which excludes dissolution or GBS to produce them, but instead involves the dynamic of dislocations. We then confirm that quartz is dominated by dislocation creep with evidence of a moderate to strong lattice-preferred orientation (LPO) and numerous tilt/twist boundaries, including at the pluton margin where rocks embrittled. These features coincide with (1) randomly oriented ‘inclusion’ quartz grains along tilt/twist boundaries and (2) a partial dependency of the LPO strength on grain size. Our findings suggest that pores arise from coalescing dislocations at boundaries of rotating sub-grains, providing nucleation sites for new grains to be precipitated during plastic flow. Fluid infiltration, rock embrittlement and related implications are also expected through pores accumulation with increasing strain.

## Introduction

In deep environment, typically at temperatures that exceed half of the rock melting point, the capacity of grains to change their shape upon deformation might prevent the persistence or production of any porosity. However, frequent documentations of fluid inclusions, nominally-hydrated minerals and/or dissolution–precipitation processes attest to fluid-rock interactions in the viscous/ductile realm, particularly in shear zones where strain has been localized^1^. This necessarily involves some mechanisms—related or not to deformation—to produce a connected porosity, so that fluids can infiltrate the rock and influence geological processes.

The production of micro-pores in sub-solidus viscous rocks has been under-investigated until the end of the 2000’s. X-ray micro-tomography images combined with microstructural observations have then demonstrated that transient pores may open and drive fluids during the activity of ductile shear zones^2^. Together with evidence of micro-pores attributed to creep cavitation in crustal rocks ever since^3,4^, electron microscopy images on broken surfaces later confirmed the presence of such pores in shear zones, but rather than related to deformation, this porosity has been alternatively attributed to dissolution effects, somehow driven by dislocation densities^5,6^. The role of deformation in producing a deep porosity therefore remains an open question that we address in this paper.

When rocks deform by viscous flow, their deformation mechanisms mostly include grain-size-insensitive (GSI) dislocation creep *versus* grain-size-sensitive (GSS) diffusion creep—or equivalents—that compete with each other to accommodate ductile strain. While diffusion creep operates by migration of vacancies through the crystal lattice in combination with important grain boundary sliding (GBS), dislocation creep involves lattice-controlled crystal plasticity with limited GBS^7^. Because micro-pores are commonly documented in fine-grained, polymineralic shear zones, typically where diffusion creep is expected to be dominant^2–4^, the way that pores open at grain boundaries is today visualized as a consequence of GBS according to the model of creep cavitation^2,3,8–13^.

Nevertheless, recent tomographic images of quartz ribbons within an ultramylonitic, quartzo-feldspathic shear zone have revealed the presence of unexpected micro-pores that cannot simply be attributed to diffusion creep, and hence, to grain boundary sliding^4^. This corroborates previous observations that documented the presence of micro-pores in polymineralic shear zones dominated by dislocation creep^5^. Moreover, statistical characterizations of experimentally deformed Carrara marble have lately shown that a significant porosity may emerge with dynamic recrystallization accommodated by sub-grain rotation^14^. It seems therefore that, like diffusion creep via GBS, dislocation creep might be involved to produce micro-pores via crystal plasticity. However, although significant implications are expected for the brittle-ductile transition and fluid circulations in deep rocks^2,15^, a porosity induced by plastic flow remains to be evidenced to occur in natural rocks.

In this study, we describe microstructural features of quartz-rich shear bands that developed within the Naxos western granite below the central-Cycladic detachment (Aegean Sea, Greece). Combining backscattered electron images and detailed electron backscatter diffraction (EBSD) maps, we highlight sub-micron pores at grain and intra-grain boundaries of pure quartz layers, giving rise to a link between porosity and dislocation motion. Together with EBSD features of quartz aggregates that indicate dislocation creep to be dominant, as well as new grains to nucleate into porphyroclasts, this strongly suggests that micro-pores result from crystal plasticity and related dislocation dynamics that we discuss in the context of the Naxos western granite.

## Results

### The strain gradient of the Naxos western granite

**Figure 1:**
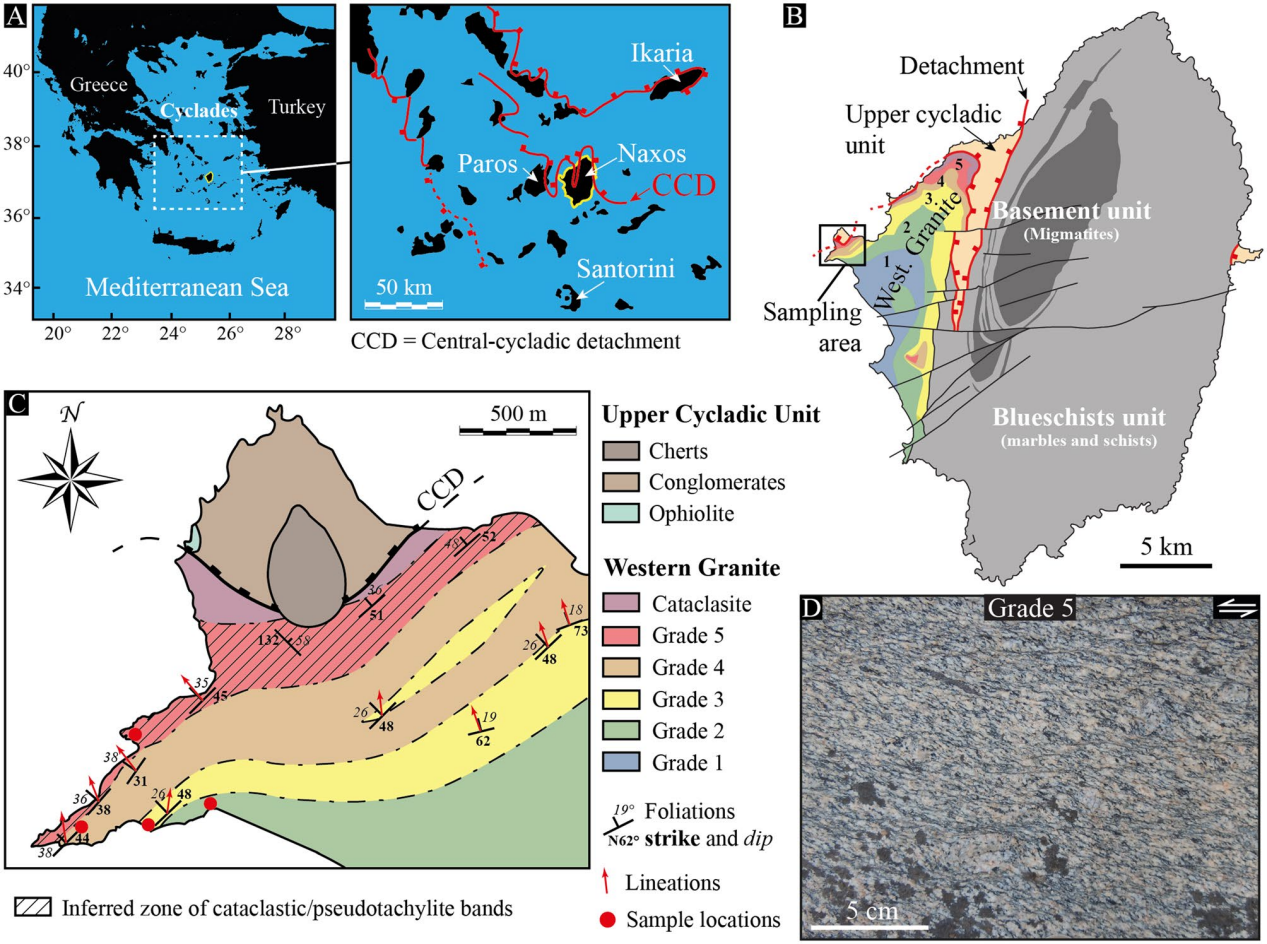
The Naxos strain gradient. (**A**) Location of the Naxos Island in the Cyclades and its position with respect to major detachments of the Aegean domain, including the central-Cycladic one (modified after Jolivet et al.^16^). (**B**) Simplified structural map of Naxos locating the western granite of the island (modified after Bessière et al.^27^). In color are shown the deformation grades from 1 to 5, as well as the sampling area of this study. (**C**) Structural map of the sampling area, including the sample location from grade 2 to grade 5^27^. (**D**) Outcrop picture of the Naxos granite illustrating quartz-rich shear bands in grade 5. The photograph is oriented normal to the shear plane and parallel to the shear direction (X–Z structural plane) with top-to-the-left kinematics.

Naxos is the largest Island of the Cyclades in the Mediterranean realm (Fig. 1A). Its geology results from a complex history related to the dynamic of the African subduction zone below the Eurasian plate. Following a period of compression over the early Cenozoic, the Aegean domain started to extend southward because of the slab rollback dynamics of the African subduction zone around 35 Ma ago, giving rise to a collapse of the mountains belt, remnants of which are observed in the present-day Rhodope, Hellenides and Taurides^16^. During extension, several low-angle normal faults, i.e., detachment faults, developed east–west along strike and unroofed deep crustal rocks within metamorphic core complexes of the resulting back-arc basin^17^. These deep materials are now below the sea level, but some of them crop out in several islands of the Cycladic archipelago and they include migmatitic domes and plutons that testify of the occurrence of partial melting during exhumation.

The Island of Naxos is one of the most complete exposure of a migmatitic dome intruded by a granitic pluton below the top-to-the-north, central-Cycladic detachment^18^ (Fig. 1B). The dome covers most of the eastern island and includes marbles, schists, amphibolites and some gneisses together with migmatites at the center^19,20^. While dating of the migmatites and related rocks yielded ages ranging between 25 ± 5 Ma and 11 Ma^19,21^, the metamorphic dome has been intruded by the granite on the western island around 12 Ma ago^19,21,22^. The paragenesis of the latter includes quartz, plagioclase, K-feldspar, biotite and hornblende with minor chlorite, titanite, apatite and oxides^23^. Local myrmekite, i.e., fine-grained intergrowth of quartz and plagioclase around K-feldspar, is also observed all across the pluton^24^. Pressure and temperature conditions of its emplacement and crystallization have been estimated around 650–680 °C and 150–300 MPa^18,22,25^.

**Figure 2:**
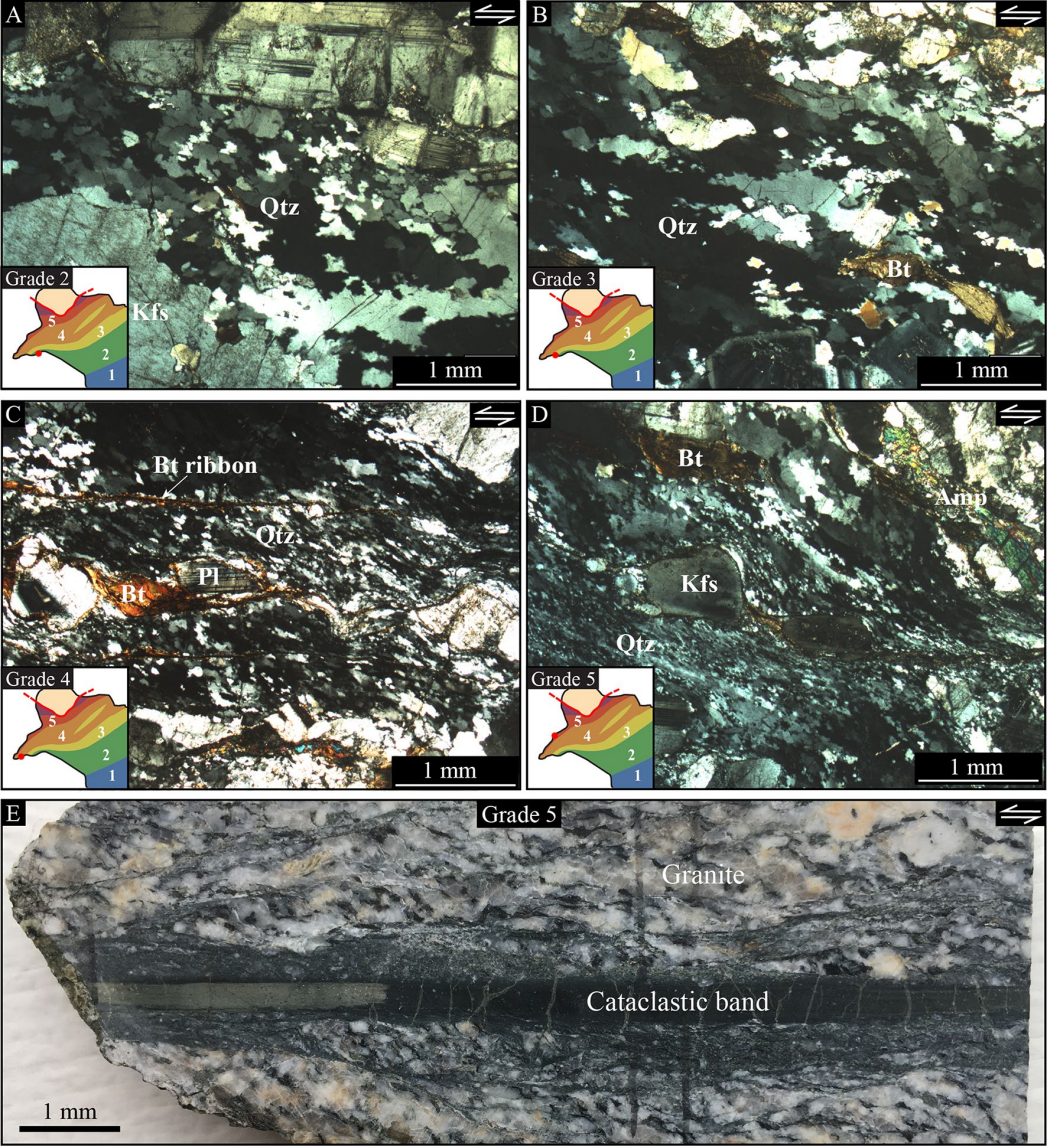
Microstructural view of quartz-rich shear bands and related features. (**A–D**) Cross-polarized optical microscopy images of shear bands from grade 2 to grade 5. The subset gives the sample location. *Qtz* quartz; *KFs* K-feldspar; *Pl* Plagioclase; *Bt* Biotite. (**E**) Hand-scale sample of a cataclastic layer from grade 5. All samples are shown in the X–Z structural plane with top-to-the-left kinematics.

During final emplacement and crystallization, the pluton of Naxos was deformed by the central-Cycladic detachment, giving rise to a strain gradient from core to rim^25,26^. Based on macro- and micro-structural features, Bessière et al.^27^ described this strain gradient using 6 grades from magmatic conditions in the core of the pluton (grade 0) to mylonitic/cataclastic deformation (grade 5) below the detachment fault (Fig. 1B). In this study, we focus on quartz-rich shear bands from grade 2 to grade 5 (Fig. 1C,D), the microstructural features of which are summarized as follow:

1. Strain induced by the detachment is observed in grade 2 where the transposition of biotite grains highlights weakly developed quartz-rich shear bands. Quartz occurs as elongated crystals with undulatory extinction and highly lobate and irregular grain boundaries (Fig. 2A).
2. Approaching the detachment in grade 3, the granite is characterized by clasts of feldspar and deformed biotite within a matrix of quartz grains where shear bands develop with a more pronounced shape-preferred orientation. The shear bands are walled by biotite ribbons, but they do not interconnect. They are mostly composed by fine-grained quartz with some elongated quartz porphyroclasts with undulatory extinction and sub-grains. Lobate grain boundaries are still common, but less pronounced (Fig. 2B).
3. Quartz-rich shear bands are significantly developed in grade 4. They show substantial grain size reduction, locally producing typical mylonites with a fine-grained matrix. Quartz grains with strong undulatory extinction and sub-grains are common, together with biotite ribbons that further develop along the shear bands (Fig. 2C). Some larger quartz crystals still occur with lobate boundaries and pronounced undulatory extinction. A few shear bands also developed where the myrmekite is observed.
4. Closer to the detachment in grade 5, the granite is highly deformed. The remaining porphyroclasts are surrounded by fine-grained shear bands, describing typical S-C (C’) fabrics (Fig. 2D). Most of the quartz-rich shear bands are mylonitic and act as interconnected layers, but they remain monomineralic, except for very limited fine-grained areas where incipient phase mixing of quartz, feldspar and biotite occurs. The biotite ribbons substantially extend and large grains of quartz have strong undulatory extinction and many sub-grains. The brittle-ductile transition definitely occurred in this grade, as shown by numerous cm-scale pseudotachylites and cataclastic layers^28^ (Fig. 2E).

### Evidence of micro-pores at quartz boundaries

**Figure 3:**
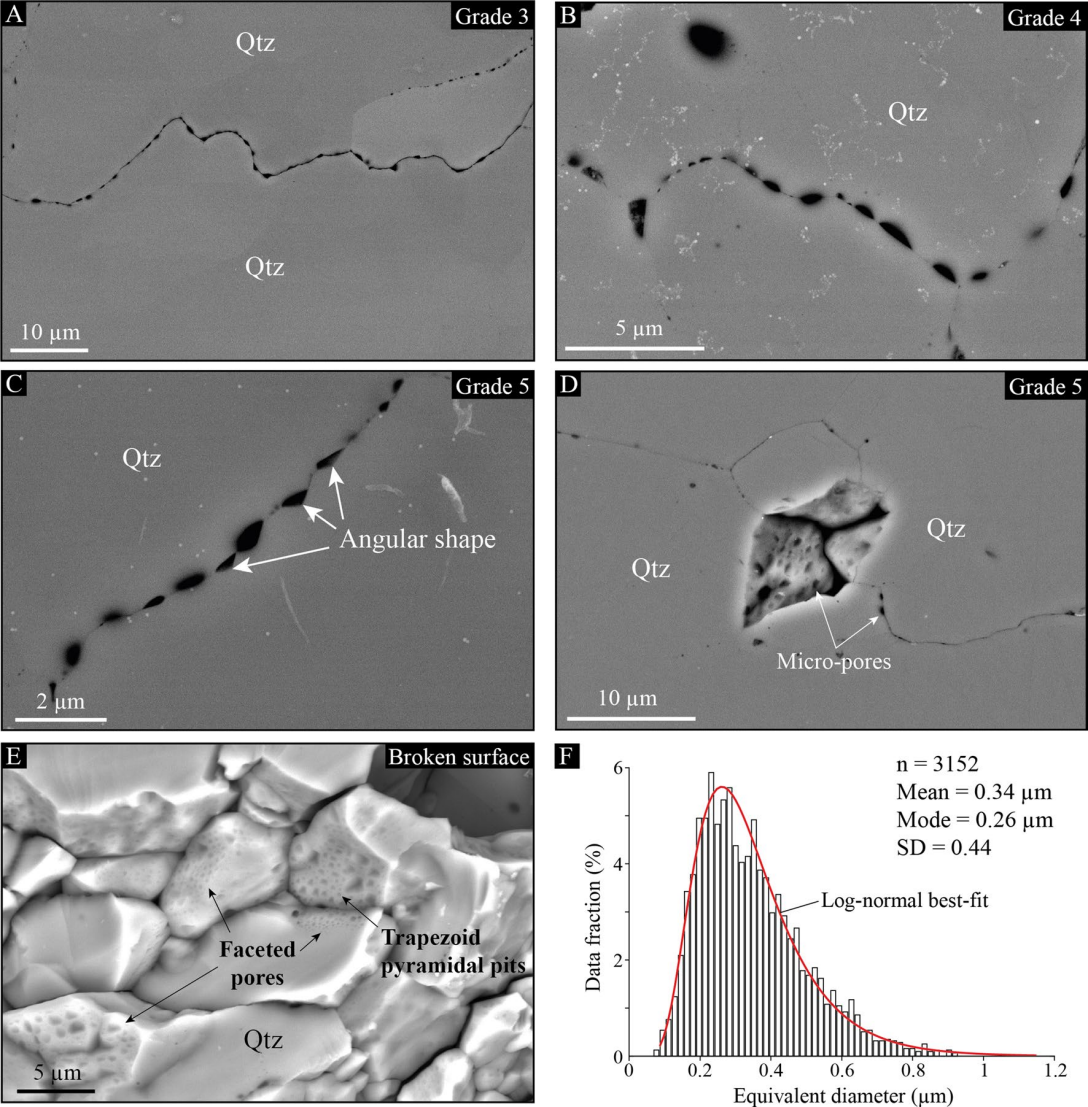
Micro-porosity in quartz-rich shear bands. (**A–C**) Backscattered electron (BSE) images locating representative areas where micro-pores have been observed at grain boundaries of grades 3, 4 and 5. These observations include cordons of pores along lobate boundaries (**A**) with gradual sizes along the boundary (**B**) and/or partly angular shapes (**C**; white arrows). (**D,E**) BSE images of micro-pores highlighted in three dimensions either through a polished surface at the favor of a detached grain during sample preparation (**D**) or on a broken surface in the YZ structural plane (**E**). (**F**) Distribution of pore sizes (equivalent diameter) as documented using pixel counting on BSE images. *n* number of pores; *SD* standard deviation.

Using high-resolution backscattered electron (BSE) images in several shear bands over the pluton of Naxos, we highlight numerous sub-micron pores at the boundaries of quartz grains (Fig. 3). These pores occur in all deformation grades, but they mostly arise in high-strain, pure quartz shear bands at grade 5. They are systematically observed along boundaries, including both, straight and lobate ones (Fig. 3A), sometimes with a gradual distribution of their sizes along the boundary (Fig. 3B) and/or partly angular shapes (Fig. 3C). On broken surfaces of quartz aggregates, micro-pores decorate a significant amount of—but not all—grain boundaries (Fig. 3D), where faceted pores may occur as trapezoid pyramidal pits at equidistance from each other (Fig. 3E). Their size ranges from less than 100 nm to no more than 1 µm with a mean size of 0.34 µm two-dimensional equivalent diameter. The mode and standard deviation have been also estimated as respectively equal to 0.26 and 0.44 µm using a log-normal best-fit distribution of the size dataset (Fig. 3F).

**Figure 4:**
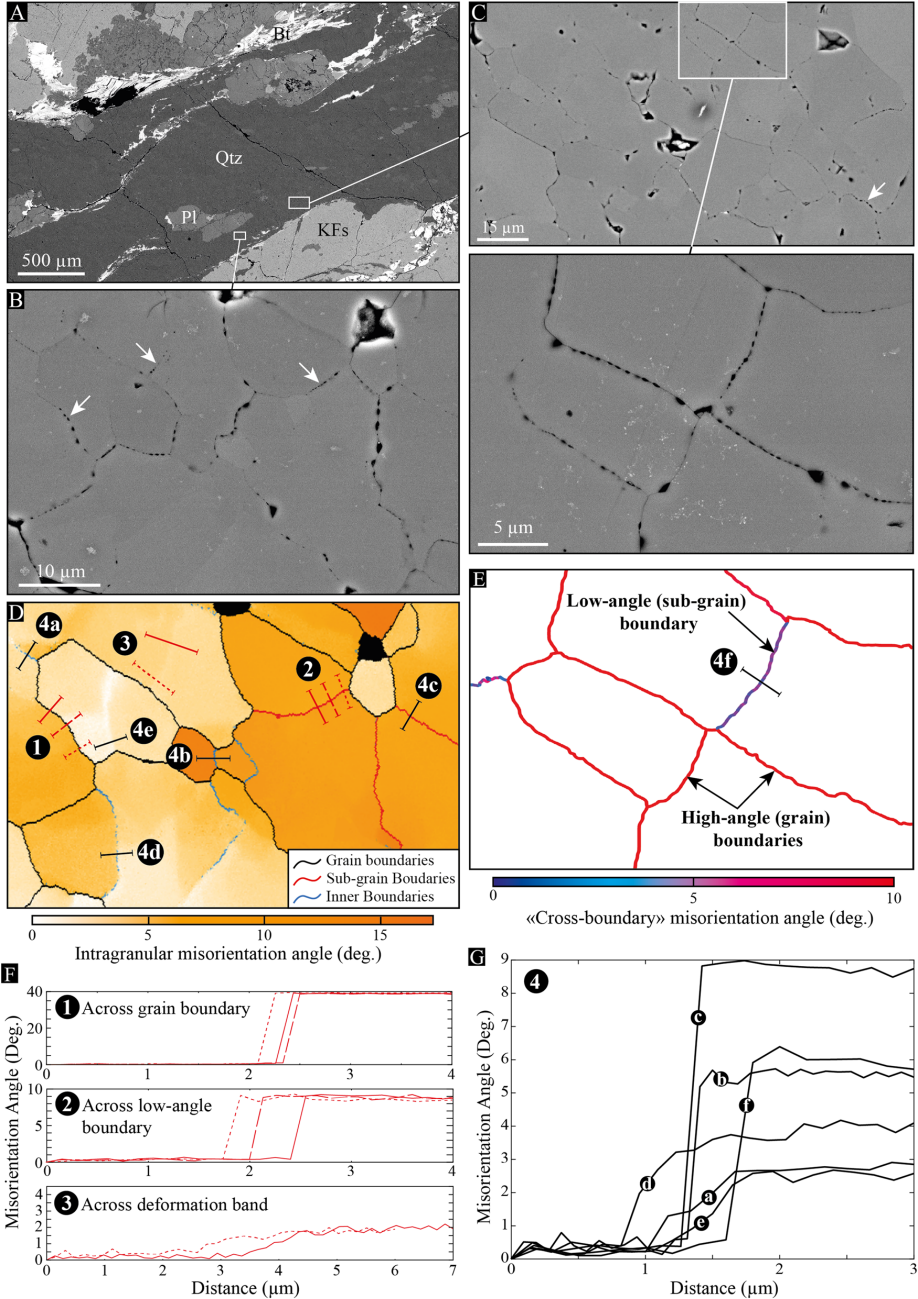
Micro-pores *versus* quartz boundaries. (**A–C**) BSE images of a quartz-rich shear band in grade 4, where the distribution and size of micro-pores have been compared to lattice misorientations using EBSD maps. White arrows indicate further examples of micro-pores, the size of which gradually increases (or decreases) along the boundary. (**D,E**) EBSD maps that documents intragranular misorientations (orange color bar) and “cross-boundary” misorientation angles (blue/red color bar) across the areas shown in (**B**) and (**C**), respectively. In (**D**), grain (misorientation angle > 10°), sub-grain and inner (misorientation angle between 2 and 10°) boundaries are respectively shown in black, red and blue. (**F**) Transects across three types of boundary: (1) grain boundary; (2) low-angle boundary; and (3) deformation band, all located in (**D**) using either solid or dashed lines. (**G**) Compilation of transects across low-angle boundaries and deformation bands located in (**D**) and (**E**). Although these boundaries describe misorientations ranging from 2° to almost 9°, all of them are decorated by micro-pores, regardless of the boundary type.

To better characterize the distribution of these pores with respect to plastic features of quartz grains, we acquired EBSD maps with a very fine step size (1/4 µm) over two areas in grade 4 where numerous micro-pores have been observed, including some of them as small as a few tens of nanometers (Fig. 4A-C). Most of the micro-pores align with high-angle (grain) boundaries, but a significant part of them decorates intra-grain boundaries, such as low-angle (sub-grain and inner) boundaries and deformation bands, i.e., planes of a weak and progressive lattice rotation over several pixels (Fig. 4D,E). In Fig. 4F, we further provide a set of transects that document lattice misorientations across different types of boundary, including (1) a grain boundary (misorientation > 10°), (2) a low-angle boundary (misorientation between 2 and 10°) and (3) a deformation band (weak and progressive misorientation). In any case, the misorientation angle remains the same along each boundary, even where micro-pores distribute with gradual sizes (arrows in Fig. 4B,C). Moreover, we provide additional transects along low-angle boundaries and deformation bands in Fig. 4G (located in Fig. 4D,E). They describe misorientation angles that range from less than 2° to more than 9°, including misorientations that spread over more than 1 micron (deformation bands). This confirms the presence of micro-pores along intra-grain boundaries and not only at grain boundaries.

### EBSD features of quartz-rich shear bands

**Figure 5:**
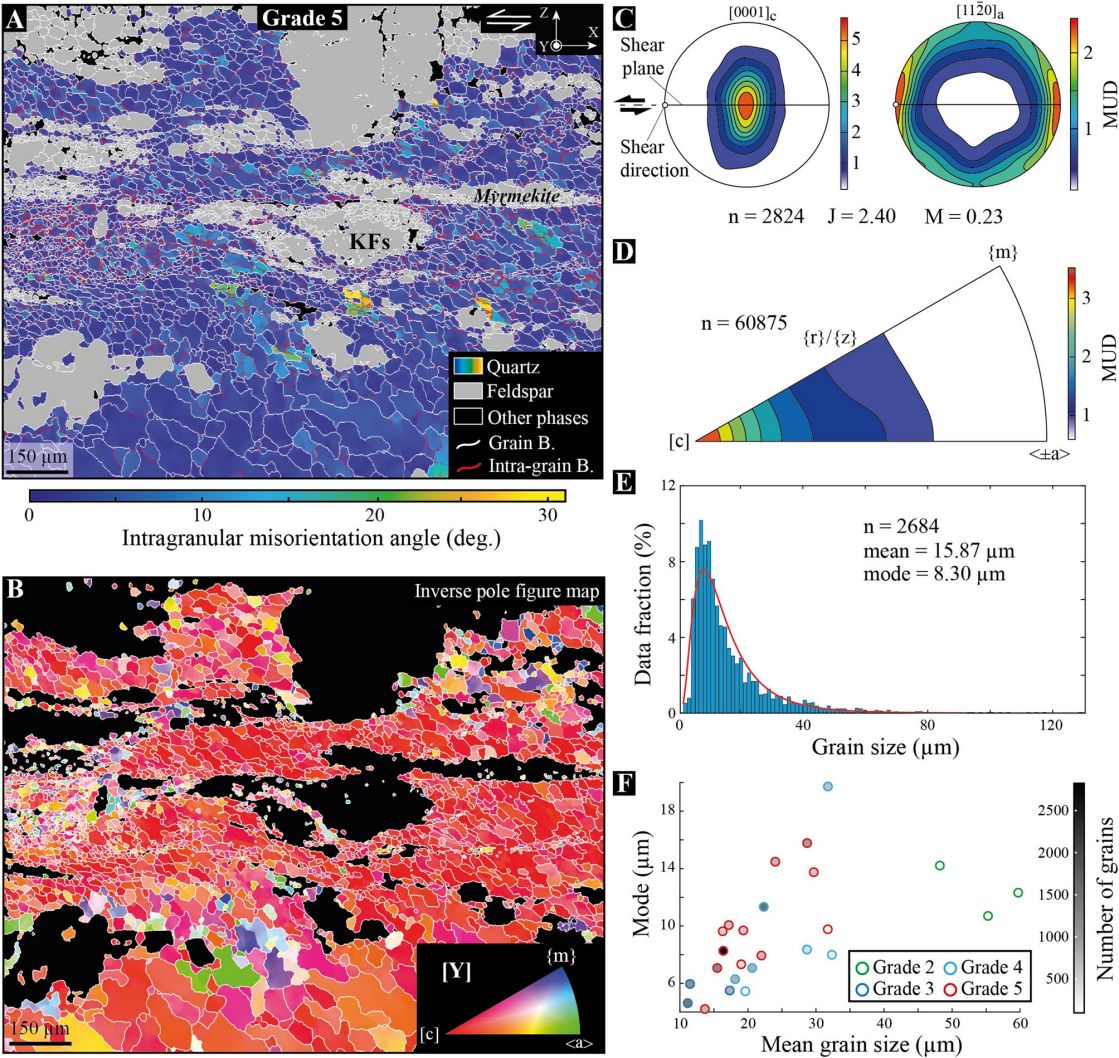
EBSD features of quartz-rich shear bands. (**A**) Intragranular misorientations of quartz grains across a shear band from grade 5 in the X–Z structural plane with top-to-the-left kinematics. This map gives the misorientation angle of one ‘pixel’ orientation within a given grain with respect to the mean orientation of the same grain (‘mis2mean’ function in MTEX^54,55^). While grain boundaries (B.) are shown in white, intra-grain ones are displayed in red. *KFs* K-feldspar; *X* shear direction; *Y* cardinal axis; *Z* pole to shear plane. (**B**) Inverse pole figure map of the same area with respect to the cardinal axis ([Y]). The color scheme of the inverse pole figure (subset) gives the crystallographic axis that parallelizes with the [Y] axis for a given data point in quartz grains. With increasing strain in the shear band center, quartz grains tend to concentrate their [c] axes around [Y]. (**C**) Lattice-preferred orientation of quartz for the area shown in (**A**) and considering the mean orientation of each grain. Distributions of the [0001]_c_ and [11$\overline{2 }$0]_a_ axes are shown in lower-hemisphere, equal-area pole figures using iso-contours and a color bar to highlight multiples of a uniform distribution (MUD). *n* number of grains; *J* texture index^56^; *M* misorientation index^32^. (**D**) Distribution of misorientation axes across low-angle boundaries shown in (**A**). As highlighted by multiples of uniform distribution (MUD), most axes concentrate around the [c] axis, indicating dominant prism-$<$a$>$ dislocation slip-system to produce intra-grain boundaries (see text). *n* number of axes. (**E**) Grain size distribution for quartz shown in (**A**). Grains cut by the map frame are excluded from the dataset. The mode is estimated using a best-fit (‘histfit’ function in Matlab) log-normal distribution (red curve). *n* number of grains. (**F**) Compilation of means and modes of quartz grain size collected from EBSD maps in shear bands of the western granite. While the grey shading bar indicates the number of grains in each map, color circles refer to the deformation grade.

From grade 2 to grade 5, a total of 31 EBSD maps have been acquired across several shear bands of pure quartz aggregates. Among representative ones (supp. Fig. 1), we provide in Fig. 5 an example of EBSD map from grade 5 (see the ‘Method’ section for details about EBSD acquisitions). This map describes strong intragranular misorientations of quartz grains, which may locally exceed 30°, particularly where strain has been localized (Fig. 5A). This always coincides with grain size reduction and an increase in low-angle boundary density, together with an overall clustering of the quartz [c]-axes around the cardinal axis ([Y]) with increasing strain (Fig. 5B). The occurrence of myrmekite that develops around K-feldspar is also observed, but not systematically related to the development of shear bands.

Accordingly, quartz lattice- (or crystallographic-) preferred orientation (LPO) concentrates the [0001]_c_ axes around [Y] and < 11$\overline{2 }$0 >_a_ axes around 3 points maxima distributed normal to [Y] at 60° from each other. While these $<$a$>$ maxima more or less smear out depending on the grade and shear band area^27^, the strongest one always tends to align with the lineation [X] (Fig. 5C and supp. Fig. 2). The distribution of [c] axes also describe half of a girdle that extends towards the [Z] axis and may locally give rise to two maxima on each side of the [Y] axis (supp. Fig. 2). This fabric is typical of a Y-maxima LPO^29^ and characterizes most of quartz-rich shear bands all over the pluton^27^. Its strength ranges from moderate to strong within the shear bands with J_indices_ higher than 2 and M_indices_ higher than 0.2 (J and M are defined in the ‘Method’ section). In relation to this, low-angle boundaries are related to lattice ‘cross-boundary’ rotation axes that mostly cluster around the [c] axis, indicating dominant slip of dislocations on {m} plane in the $<$a$>$ direction (Fig. 5D), also referred to as prism-$<$a$>$ slip system^30,31^. The grain size always shows a log-normal distribution (Fig. 5E) and decreases overall up from grade 2 (supp. Fig. 2), but the mean grain size (and mode) strongly varies between ~ 10 and ~ 30 μm (between 4 and 20 μm for the mode) from one shear band to another, irrespective of the grade (Fig. 5F).

**Figure 6:**
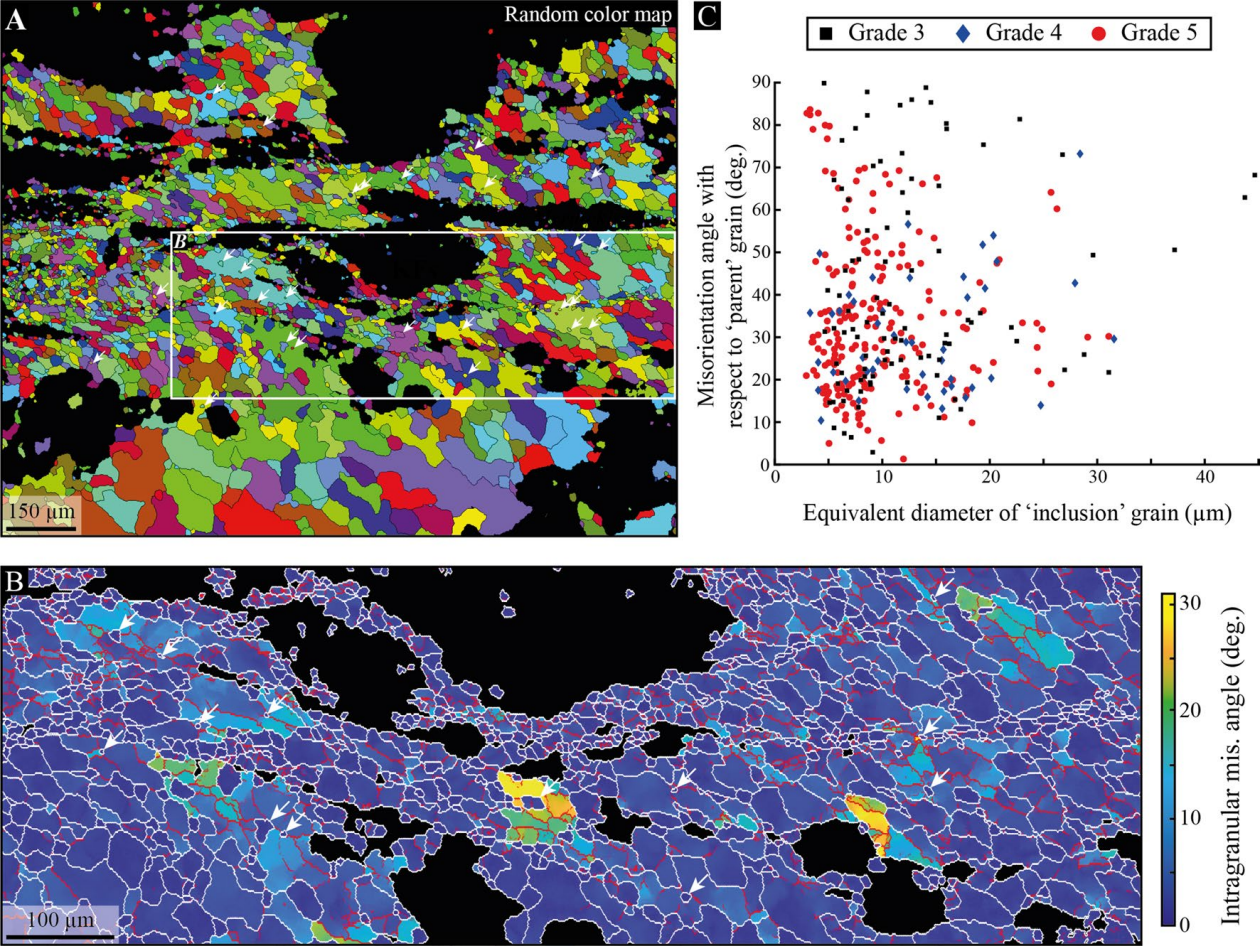
‘Inclusion’ quartz grains. (**A**) Random colors attributed to each quartz grain from the map shown in Fig. 5A. This map highlights small isolated grains (white arrows) within larger ones. (**B**) Intragranular misorientation map of the area shown in (**A**). White arrows indicate isolated ‘inclusion’ grains that all locate at low-angle boundaries (red lines). Grain boundaries are shown in white and the color bar refers to intragranular misorientation angles. (**C**) Grain size (equivalent diameter) *versus* misorientation angle of ‘inclusion’ quartz grains with respect to their parent one, as collected from EBSD maps performed in grade 3, 4 and 5 (further examples of inclusion grains are given in supp. Fig. 3).

In addition, the presence of numerous quartz grains was found as ‘inclusion’ into larger ‘parent’ ones (Fig. 6A and supp. Fig. 3). They arise in shear bands of all grades with various sizes and systematically along intra-grain boundaries (Fig. 6B). By compiling data from several shear bands of grades 3, 4 and 5, we show that the misorientation angle of these ′inclusion′ grains can be as high as 90° regardless of the grade or grain size, which ranges from 3 µm (i.e., the detection limit imposed by the 1-µm step size of EBSD maps) to more than 30 µm with a large part of them below 10 µm (Fig. 6C).

### LPO versus grain size

**Figure 7:**
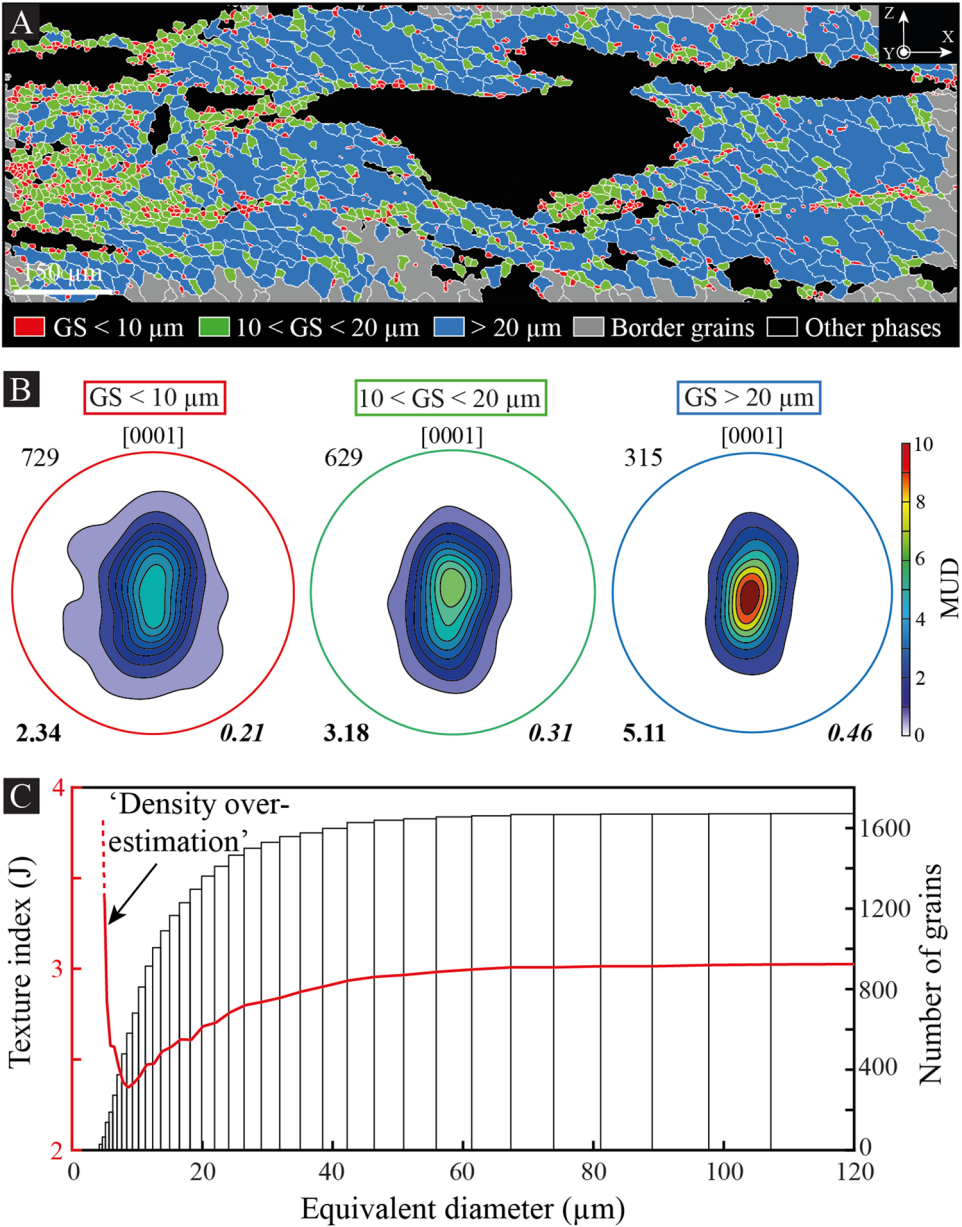
Texture strength dependency on grain size. (**A**) EBSD map of the shear band from grade 5 shown in Fig. 5A,B. Only quartz of the shear band center is shown for three categories of grain size (GS; equivalent diameter): (1) lower than 10 μm; (2) between 10 and 20 μm; and (3) above 20 μm. Grains cut by the map frame (i.e., borders grains) and grains composing myrmekite areas are not considered. *X* shear direction; *Y* cardinal axis; *Z* pole to shear plane. (**B**) Lattice-preferred orientation of each class of grain size. Only the [0001]_c_ axis is shown in lower-hemisphere pole figures, together with the number of grains (top left in regular), texture (J) index (bottom left in bold) and misorientation (M) index (bottom right in bold italic). *MUD* multiples of uniform distribution. (**C**) Equivalent diameter *versus* J_index_ for several populations of quartz grains sorted by ascending grain size and using a cumulative approach (the same graph using M_index_ is available in Supp. Fig. 4). The histogram gives the number of grains in each class. As highlighted in (**B**), the J_index_ distribution using a cumulative approach confirms a positive dependency on grain size, except below 8 μm where J describes an abrupt opposite behavior due to density overestimations (see text and ‘Method’ section).

In several shear bands of the granitic pluton, our EBSD maps and documentation of grain size and texture/misorientation indices have revealed a dependency of the fabric (or texture) strength on grain size. This feature—mostly observed in grade 5, but also in grade 4—is illustrated in Fig. 7, where quartz LPOs have been plotted for three categories of grain size (equivalent diameter): (1) lower than 10 µm; (2) between 10 and 20 µm; and (3) larger than 20 µm (Fig. 7A,B). This excludes ‘border’ grains, i.e., grains partially cut by the map frame, and quartz grains in myrmekite. We thus used both, the texture (J) and misorientation (M) indices to calculate the strength for each category, giving rise to LPOs that significantly strengthen with increasing grain size from J = 2.34/M = 0.21 below 10 µm to J = 5.11/M = 0.46 above 20 µm (Fig. 7B). We also made sure that a significant number of grains was selected. Indeed, because of density overestimations through calculation of J and M, both indices may be artificially dependent on the number of discrete data^32^. Based on olivine datasets, a minimum of 150 grains has been proposed to avoid this artefact and calculate a relevant fabric strength^32^, but this number is questionable^33^ and may change depending on the crystal symmetry and intensity of LPO. Hence, we developed a new method to detect the threshold number above which density overestimations are not effective anymore to calculate texture strength. This implies using a map dataset to calculate the J_index_ (or M_index_) for several classes of grain size, but for many more grain size categories by considering a cumulative number of grains sorted by ascending grain size (Fig. 7C). This procedure does not allow to access the respective fabric strength of each class, but the number of grains remains significant for most categories and we see how the J_index_ evolves by adding new grains to the growing dataset (see the ‘Method’ section for further details). In Fig. 7, our method confirms a significant increase of J_index_—or M_index_ (see supp. Fig. 4)—with increasing equivalent diameter, except below 8 µm where the J_index_ drops drastically. The density overestimation threshold is here estimated to be ∽500 grains.

**Figure 8:**
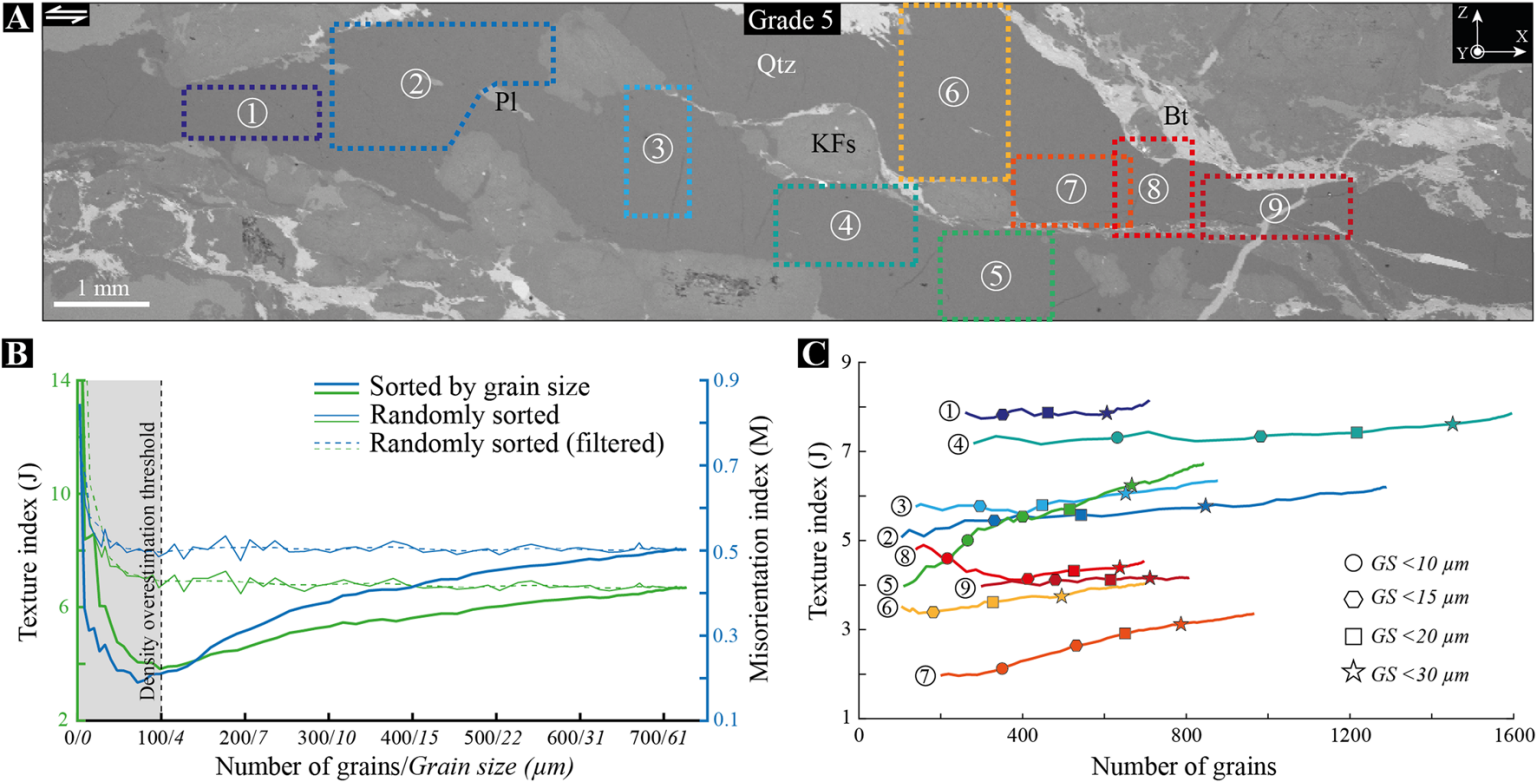
Texture strength dependency on grain size (continued). (**A**) BSE image of a shear band in grade 5 where nine EBSD maps are located (EBSD maps are available in Supp. Fig. 5). *X* Shear direction; *Y* cardinal axis; *Z* pole to shear plane; *KFs* K-feldspar; *Qtz* quartz; *Pl* Plagioclase. (**B**) Texture (J) and misorientation (M) indices *versus* number of grains/grain size extracted from the EBSD map #5 and located in (**A**). J and M are calculated for a cumulative number of grains either sorted by ascending grain size or randomly selected in the same dataset (randomly sorted). This graph exemplifies our procedure to define the threshold number of grains (here 100 grains) above which J and M are relevant, i.e., independent on density overestimations (see the ‘Method’ section). The dashed line refines the threshold value by applying a zero-phase digital filtering to J and M based on randomly sorted data. (**C**) Texture (J) index *versus* number of grains extracted from all maps shown in (**A**). J is calculated for a cumulative number of grains sorted by ascending grain size. Only data above the density overestimation threshold have been considered. Symbols give the J_index_ and number of grains for grain size (GS) populations lower than 10, 15, 20 and 30 μm. While most EBSD maps highlight a positive dependency of the fabric strength on grain size, this dependency weakens or even disappears for some of them (see text).

To better document the dependency of the fabric strength on grain size, we compiled datasets from several EBSD maps acquired over a larger shear band in grade 5 (Fig. 8 and supp. Fig. 5). For each map in Fig. 8A, the J_index_ has been calculated as a function of grain size considering categories with a cumulative number of grains, as described in Fig. 7C and the ‘Method’ section. We further checked for density overestimations through calculation of the J and M indices based on the same number of grains for each category, but selecting grains randomly—rather than sorted by size—in the same dataset (see the ‘Method’ section). This gives rise to a threshold number of grains above which the fabric strength remains quasi-unchanged, and below which J and M depend on the number of discrete data, as exemplified for map #5 in Fig. 8B. Only data above the density overestimation thresholds have been therefore considered and plotted in Fig. 8C, where a dependency of the fabric strength on grain size is documented for most aggregates of the shear band, particularly in maps #5 and #7. However, this dependency is differently pronounced and non-systematic, as shown in maps #1, #8 and #9 for which no dependency is observed (Fig. 8C).

## Discussion

During deformation of the western granite in Naxos, strain primarily localized into quartz-rich aggregates while the pluton was progressively cooled and uplifted. The presence of a significant quartz LPO in all shear bands, together with numerous low-angle boundaries and strong lattice misorientations, indicate that dislocation glide and climb, i.e., dislocation creep, actively contributed to deformation. The systematic occurrence of Y-maxima LPO and related governance of prism-$<$a$>$ dislocation slip-systems strongly suggest dislocation creep to be dominant all over the pluton, with a transition occurring from grain boundary migration in grade 2 to sub-grain rotation in grade 5 to accommodate plastic flow. Following the nomenclature of Hirth and Tullis^34^, this would correspond to regime 3 that progressively evolves to regime 2 with decreasing temperature and increasing strain rate across the pluton, in agreement with deformation experiments.

Of further interest is the presence of micro-pores at grain and intra-grain boundaries of pure quartz aggregates. So far, the presence of micro-pores in deep rocks has been described and/or proposed to occur during viscous creep of polyphase, fine-grained ductile shear zones^2–4,9,10^. This involves rocks to be deformed at conditions where diffusion creep—or equivalent—is predicted to be the dominant process, which is definitely not the case in Naxos. Available flow laws for quartz do not predict either GSS creep to be dominant for a mean grain size that far exceeds 1 µm^35–37^. In relation to this, similar micro-pores have been described in naturally deformed rocks dominated by dislocation creep^4,5^, but only at specific grain boundaries and aligned with dislocation lines^5^. This led several authors to propose that pores may have originated from fluid infiltration through preferential dissolution of grain boundaries where dislocations have been accumulated^5,6^. However, fluid flow is not expected to occur along sub-grain walls, and hence, our evidence of micro-pores along low-angle boundaries does not support dissolution effects as a source of porosity. This does not particularly account for micro-pores aligned with deformation bands, which are not “true” boundaries yet. Dissolution effects as promoting a log-normal distribution of pore sizes, which implies a dynamic process to equilibrate and limit them below 1 μm, is also very unlikely. Some “healed cracks” processes (fluid inclusions) may be envisaged, but (1) a healed crack does not necessarily result in a lattice misorientation, and (2) if misorientation is present, we expect a sharp one and not a progressive one, as typically observed for deformation bands.

**Figure 9:**
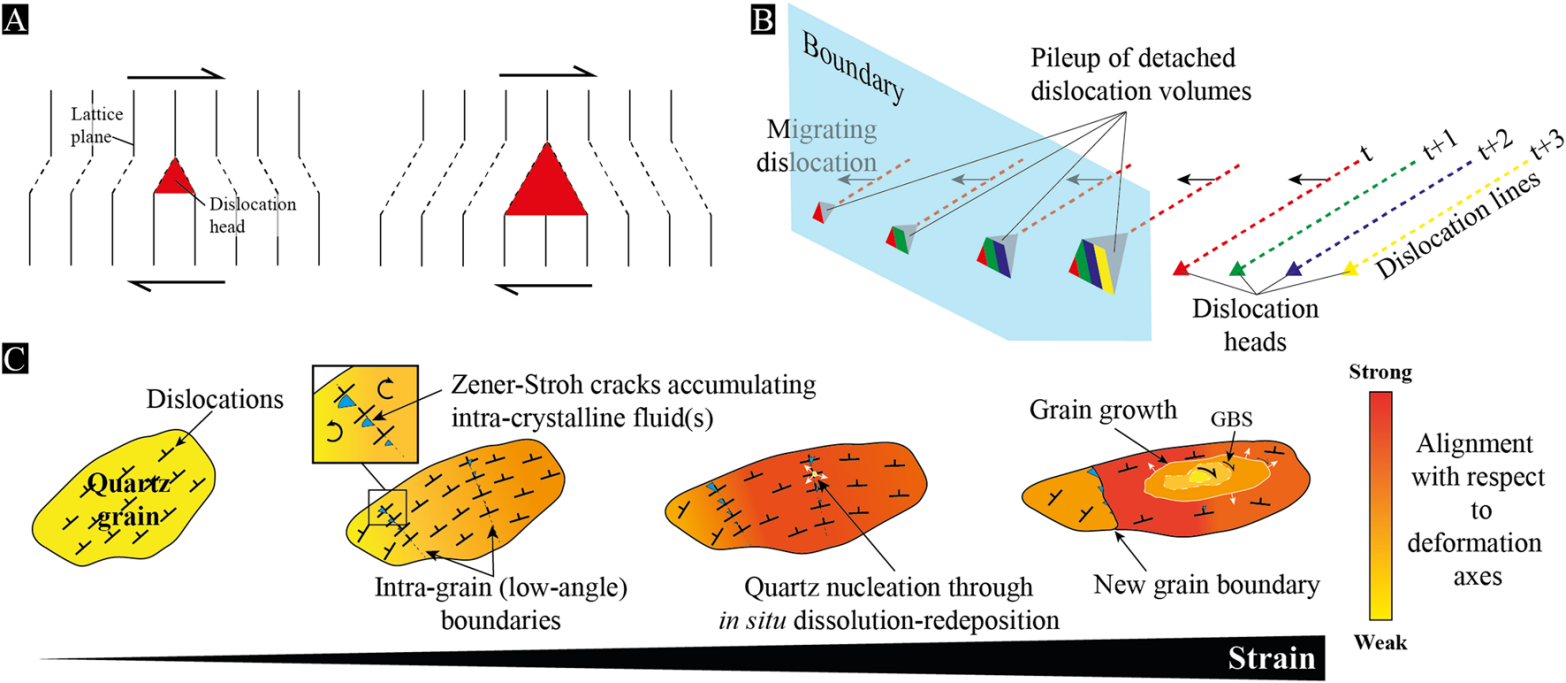
Model of micro-pores induced by coalescing dislocations during sub-grain rotation. (**A**) Mechanism of Zener-Stroh cracking to produce micro-pores by coalescing dislocations (modified after Weertman^38^). (**B**) Sketch of migrating dislocations that stop at an interface, here envisaged as a low-angle boundary, to produce micro-pores with lattice-controlled geometries and gradual sizes along the boundary. The color scheme refers to dislocation heads that coalesce and produce growing micro-pores at several time (t) increments. (**C**) Cracking and phase nucleation induced by sub-grain rotation during plastic flow of pure quartz aggregates. While quartz deforms by crystal plasticity, low-angle boundaries arise from a first dislocation slip-system, giving rise to sub-grains that rotate to produce micro-pores through intersection with a second slip-system. In this model, pores open by coalescing dislocation along a low-angle boundary until complete recrystallization of the sub-grain. Randomly oriented quartz grains are also expected to nucleate through in situ dissolution-redeposition^45^ from the collected intra-crystalline fluid into pores. New grains then subsequently rotate (GBS) and deform while they grow to align with the deformation axes. We further anticipate micro-pores to accumulate at grain boundaries, leading to rock embrittlement.

Alternatively, Gilgannon et al.^14^ recently used a statistical approach to highlight micro-cavities emerging during dynamic recrystallization of experimentally deformed Carrara marble. They proposed that micro-pores may arise from sub-grain rotation with many implications, but without expanding much on the mechanism itself. Our evidence of micro-pores at low-angle boundaries and deformation bands strongly suggests that the porosity in Naxos arose from dislocation dynamics, which is compatible with the idea of sub-grain rotation to produce it. However, according to the model of creep cavitation induced by dislocation tangles, also referred to as Zener-Stroh mechanism, micro-pores nucleate by coalescence of migrating dislocations that stop at an obstacle, such as a grain boundary^8,38^ (Fig. 9A). Sub-grain boundaries may act as interfaces to stop dislocations to migrate, but having both coalescing dislocations and a sub-grain wall resulting from the same dislocation slip-system is very unlikely; dislocations of a single slip-system either climb to produce a tilt wall or coalesce to open a pore, but they cannot contribute to both processes simultaneously.

We nevertheless envisage that micro-pores may result from the intersection of several slip-systems^39^, which is very likely to occur in any crystal that deforms plastically, particularly in the dislocation creep regime. In this view, one slip-system produces a tilt/twist wall, creating an interface along which a sub-grain is rotating, and a second slip-system interferes with the tilt/twist boundary and coalesces dislocations to produce micro-pores. This may account for pores occurring with lattice-controlled geometries (i.e., pileup of dislocation volumes), as well as with gradual sizes, if we assume that dislocations detach part of their volume at successive times to coalesce along the boundary as they migrate (Fig. 9B). Moreover, because pore opening stops after complete recrystallization of sub-grains, micro-pores are finally stored into the newly formed grain boundaries with a limited size (below 1 μm in the case of Naxos). Minor changes are nonetheless expected to occur subsequently to dynamic recrystallization if some grain boundary sliding is involved. We do not either exclude Zener-Stroh cracks to nucleate at grain boundaries, but, unlike to low-angle boundaries, their mobility—mostly driven by dislocation densities—is expected to prevent (or limit) the production of pores along them. Fluid flow and related dissolution effects are neither excluded, provided that pores interconnect with each other at grain boundaries.

In a context of creep cavitation, phase nucleation is an additional process to be considered during viscous flow of naturally deformed rocks, particularly where strain has been localized in the presence of fluids. Strain-induced cavities are indeed potential sites for new grains to be produced^3,4,9,12,14,40^. In Naxos, we highlighted numerous quartz grains arising as ‘inclusions’ along low-angle boundaries of quartz porphyroclasts. This can be an effect of strain-induced mechanisms to produce recrystallized grains^41^, but the misorientations of dynamically recrystallized grains has been documented as rarely exceeding 30° with respect to their parent one^42^. This definitely contrasts with the misorientations of the ‘inclusion’ grains that we document between 30° and 90° for a large part of them, irrespective of grain size and deformation grade. We therefore suggest that ‘inclusion’ grains might arise from a fluid phase nucleating them into micro-pores of low-angle boundaries, which would account for their (1) random orientations, (2) various sizes and (3) aligned positions with sub-grain walls and deformation bands. The production of new grains randomly oriented that subsequently align as they grow also accounts for a positive dependency of the fabric strength on grain size (Fig. 9C). Because nucleated grains necessarily mixed with dynamically recrystallized ones in various amount with respect to each other, such a grain size dependency on fabric strength is expected to be non-systematic and more or less pronounced.

This hypothesis however suggests that (1) molecular H_2_O can access micro-pores at low-angle boundaries and (2) nucleation is possible if phases of the same nature are involved, i.e., quartz nucleating into quartz. If a fluid film is present at grain boundaries, it may be envisaged that molecular H_2_O can migrate through a tilt/twist wall^43^, but this is very unlikely to occur within a deformation band along which single dislocations are not fully connected to each other. As an alternative, natural observations of quartz mylonite led several authors to propose that new quartz grains may arise from in situ solution-transfer and related dissolution-redeposition during plastic flow^44,45^. In this case, an external fluid is not required anymore to enter low-angle boundaries and precipitate a new grain; quartz nucleation results from dissolution enhanced by high dislocation densities at pore surfaces^46^, and then from subsequent local reprecipitation through a fluid phase^45^. Considering the latter as originating from the intra-crystalline fluid that has migrated and accumulated with dislocations into pores (Fig. 9C), our findings emphasize Zener-Stroh cracks, i.e., pores produced by accumulation and coalescence of dislocations, as ideal sites for quartz to nucleate by local dissolution-reprecipitation, and hence, without involving any external fluid. Such a process may account for recent documentations of experimentally deformed Carrara marble—monomineralic by nature –, where phase nucleation has occurred in creeping cavities produced by sub-grain rotation of calcite grains^14^.

Finally, together with creep cavitation induced by grain boundary sliding, Zener-Stroh cracking is a widely described process in industrial materials, particularly in metals^8,47–49^. Its documentation gave rise to the so-called Monktman-Grant relationship, which relates the applied strain rate to the failure of the material: the higher the strain rate, the shorter the time to failure through nucleation, growth and interlinkage of cavities^50–52^. The occurrence of strain-induced cavitation in viscous material is thus very similar to somewhat related to the brittle-ductile transition, such as recently proposed to account for the nucleation of earthquakes at the base of the seismogenic zone^15^. We thereby anticipate, within the model of micro-pores induced by sub-grain rotation, that pores may accumulate at grain boundaries through intensive dynamic recrystallization, leading to rock embrittlement and subsequent production of pseudotachylyte/cataclastic bands. Through pores interconnection before rock failure, we also expect an external fluid to infiltrate the rock and help radioactive elements, such as Radon-222^53^, to escape from it.

## Method

### Sample preparation and analytical conditions

Scanning electron microscopy (SEM) and electron backscatter diffraction (EBSD) analyzes have been performed on thin section extracted from rock chips oriented normal to the shear plane [X,Y] and parallel to the lineation [X]. Thin sections and/or rock chips have been respectively polished at the laboratory of Geoscience Montpellier and *Institut des Sciences de la Terre d’Orléans* using diamond pastes with decreasing granulometries from 3 to ¼ µm, and then followed by chemico-mechanical polishing using colloidal silica. Each sample has been coated by a carbon layer of ~ 20 nm thick for SEM observations, and then polished again with colloidal silica before adding a carbon “flash” coating of around 3 nm thick to prevent from any electron charging during EBSD acquisition. While backscattered electron images have been acquired using a Gemini I field Emission Gun from Zeiss available in Orléans (ISTO/BRGM, France), a TESCANx MIRA 3 XMU equipped with an EDAX Pegasus system, also available at the ISTO/BRGM, has been used to get EBSD data. SEM observations and EBSD analyzes have been performed at an accelerating voltage of 15 and 20 kV, and a working distance of 10 and 15 mm, respectively. The size of micro-pores has been estimated by contouring their area—subsequently converted in equivalent diameter—using the open-access software ‘imageJ’ (version 1.51; https://imagej.nih.gov/ij) on SEM images. EBSD acquisitions have been performed on thin sections tilted at 70°.

### EBSD mapping

Data acquisition and post-analyzing cleaning have been performed using the TSL-OIM softwares (OIM data collection and OIM analysis version 7.1; https://www.edax.com/products/ebsd/oim-analysis) with map “cleaning” procedure described in Précigout et al.^9,10^. We used a step size of 1 micron for each map, except for the maps shown in Fig. 4 where we applied a step size of a ¼ micron. The data were then treated and plotted using the open-source MTEX toolbox^54,55^ (version 5.7; https://mtex-toolbox.github.io) for MATLAB. The minimum angles of lattice misorientation to define high-angle (grain) and low-angle (sub-grain and inner) boundaries have been respectively set to 10° and 2°, and each grain contains a minimum of five consecutive pixels on several rows. While continuous low-angle boundaries are referred to as sub-grain boundaries, the discontinuous ones are referred to as inner boundaries. Whereas the crystal symmetry for quartz is trigonal 32 (point group 321), we chose to use a hexagonal symmetry (point group 622) to avoid artefacts related to dauphine twins, some of them being artificially produced during data acquisition; dauphine twins can be merged with parent grains, but even so, they significantly change the mean orientation of grains^29^. Lattice-preferred orientations are shown in equal-area, lower-hemisphere pole figures using a half-width gaussian angle of 10° and a resolution of 5°. The same values have been used to calculate the texture (J) index. Both the misorientation (M) index and J_index_ quantify the strength of a mineral LPO from uniform (J = 1; M = 0) to crystal-like (J = ∞; M = 1) distribution. While the J_index_ refers to the second moment of the distribution of discrete crystal orientations in Euler angle space^56^, the M_index_ is calculated based on the distribution of uncorrelated misorientation angles (here using the mean orientation of grains) with respect to their theorical uniform distribution^32^.

### Density overestimations related to J/M_index_ calculation

In field and experimental geology, several scalars are widely used to quantify the preferred orientation—or texture—of lattice crystals within a given aggregate. This includes the texture and misorientation indices, which are calculated differently, but provide the same—and very useful—information. However, both indices have been described as depending on the number of discrete data below a certain amount of them^32^. To tackle this issue, we here develop a numerical routine that defines the threshold number of grains above which J and M are relevant for a given EBSD map. At first, we calculate each scalar for a cumulative number of grains sorted by ascending grain size. For instance, we select all grains lower than 3 µm and calculate the J and M indices. We then add grains between 3 and 4 µm and calculate both indices again, and so on until considering all grains of the map dataset. Note that the grain size increment is not necessarily 1 µm, but may be adjusted to either refine the transition from one class to another (at small grain size) or avoid oversampling of categories (at large grain size). To check for density overestimation, we then use the same number of grains in each class, but selecting grains randomly in the map dataset. Through calculation of J and M, which we repeated several times for each category to refine the mean index values, we can define the number of grains above which J and M remain constant, and hence, do not depend on the amount of discrete data anymore. Zero-phase digital filtering (“filtfilt” option in Matlab) has been also applied to fit the J and M values over the full range of data, so that the threshold value is better defined (see example in Fig. 8B).

## Supplementary Information

Supplementary Information.

## Data Availability

All data source of EBSD maps and MTEX scripts are available under request to J.P. at the following address: jacques.precigout@univ-orleans.fr.
