# Supplementary material for: Porosity induced by dislocation dynamics in quartz-rich shear bands of granitic rocks
Source: Sci Rep. 2022 Apr 12;12:6141. doi: 10.1038/s41598-022-10053-x (PMC9005511; doi:10.1038/s41598-022-10053-x)
Supplement: Supplementary file 1 — Supplementary Information. [file 41598_2022_10053_MOESM1_ESM.pdf]

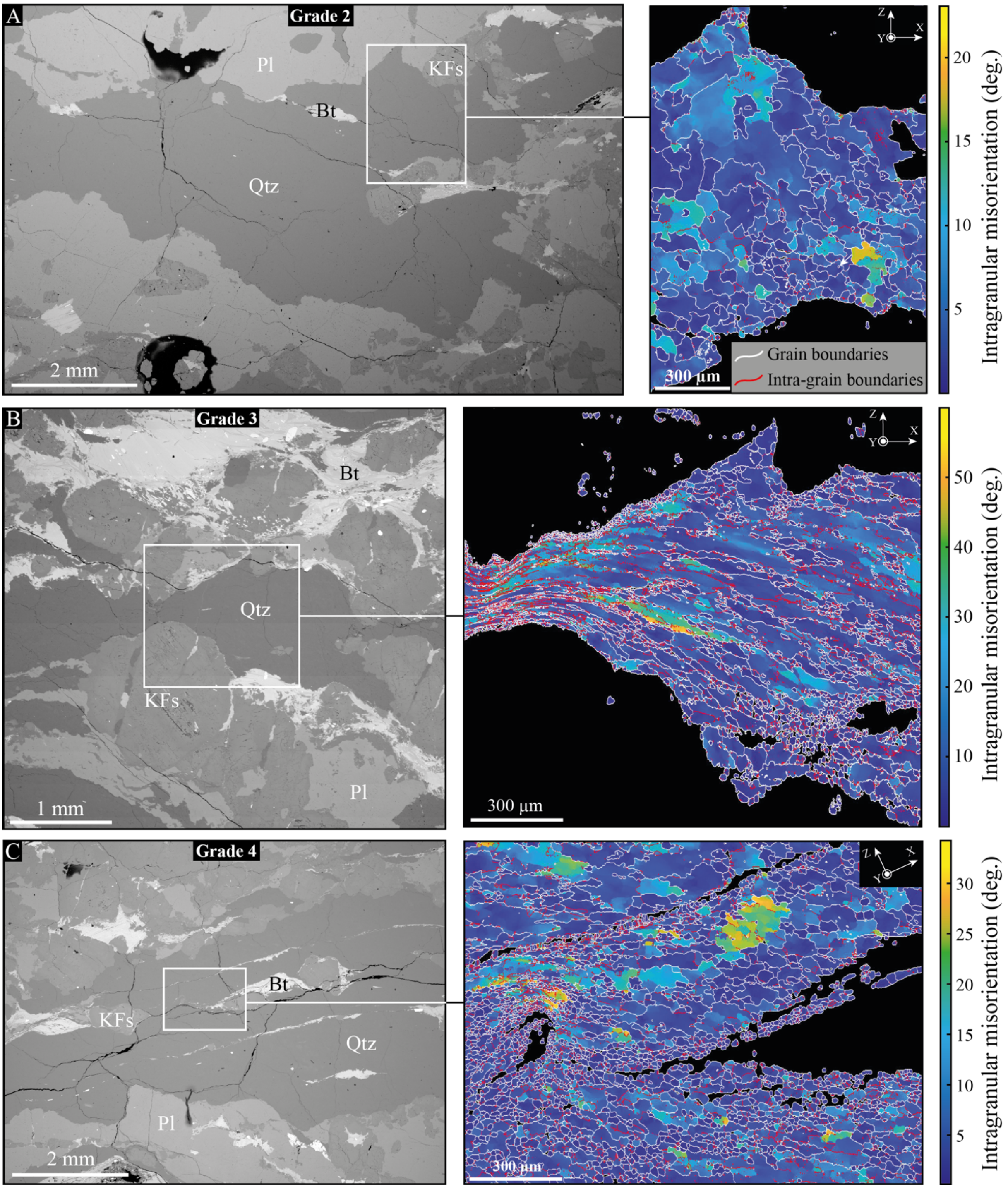

Supplementary figure 1 | Representative EBSD maps performed in pure quartz aggregates from grade 2 to grade 4. A-C) Backscattered electron images of shear bands in grades 2 (**A**), 3 (**B**) and 4 (**C**) on which EBSD maps are located. Color bars give the intragranular misorientations of quartz grains. The misorientation angle is calculated for all "pixel" orientations with respect to the mean orientation of the grain they belong ("mis2mean" function in MTEX). Grain boundaries and intra-grain boundaries are indicated in white and red, respectively. X = shear direction; Y = cardinal axis; Z = pole to shear plane; Qtz = quartz; KFs = K-feldspar; Pl = Plagioclase; Bt = Biotite.

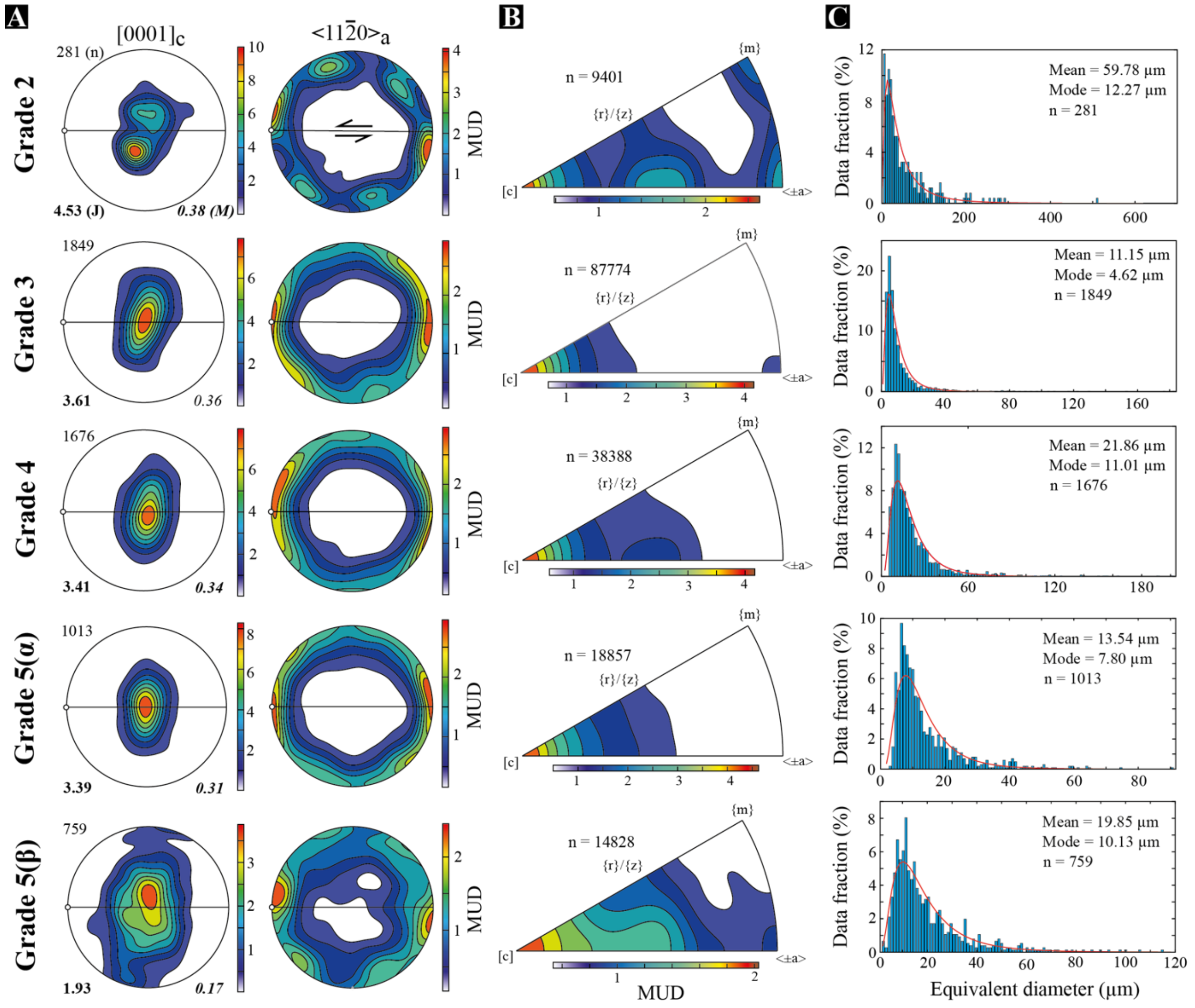

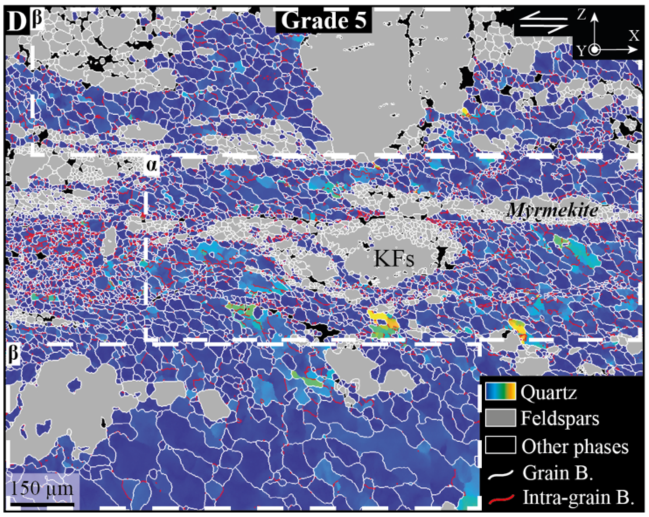

Supplementary figure 2 | EBSD features of representative shear bands from grade 2 to grade 5. A) Quartz lattice preferred orientation (LPO) of maps shown in supp. Fig. 1 and Fig. 5 of the manuscript. LPOs are given in lower-hemisphere pole figure of the [0001]c and <11$\bar{2}$0>a axes, considering one point per grain (mean orientation). Color bars and iso-contours are multiples of uniform distribution (MUD). n = number of grains; **J** = texture index; ***M*** = misorientation index. **α** and **β** refer to two areas of the map shown in **D**, one in the shear zone center and a second one that combines two areas on each side of the shear band. **B**) Distribution of rotation axes across segments of intra-grain boundaries with respect to the reference axes of hexagonal quartz. n = number of axes. **C**) Grain size distribution with best-fit, log-normal correlation. **D**) Location of the **α** and **β** areas on the map shown in Fig. 5A of the manuscript. The corresponding data are shown in **A-C**.

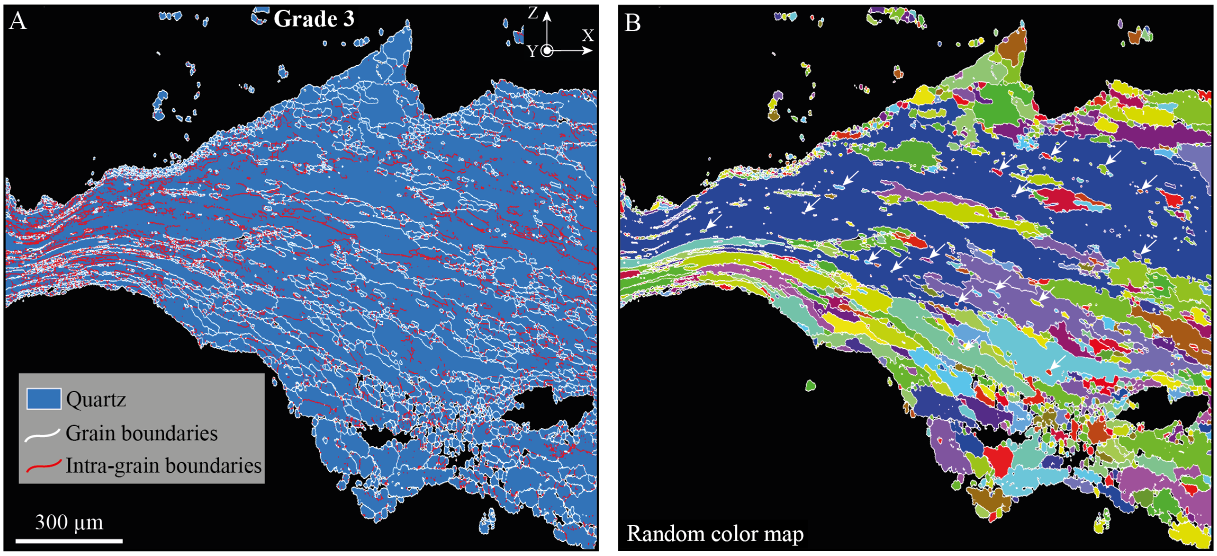

Supplementary figure 3 | 'inclusion' grains in pure quartz aggregates. A) EBSD map for quartz of a shear band in grade 3. The map is located in supplementary figure 1. Grain boundaries and intra-grain boundaries are shown in white and red, respectively. X = shear direction; Y = cardinal axis; Z = pole to shear plane. **B)** Random color map of quartz grains that compose the aggregate. One color is attributed randomly to each grain, highlighting numerous isolated grains (white arrows) into larger quartz ones and here referred to as 'inclusion' grains.

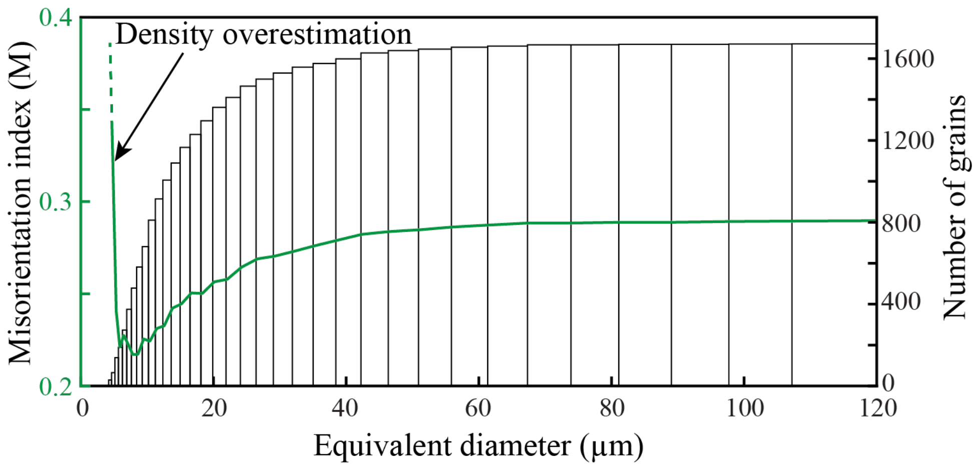

Supplementary figure 4 | Strength (M_{index}) dependency on grain size. Misorientation (M) index *versus* equivalent diameter of quartz grains sorted by ascending grain size and using a cumulative approach. The histogram gives the number of grains of each class for which we calculated the M_{index}. This supplementary figure complements the figure 7C, where the density overestimation artefact related to J/M_{index} calculation is addressed.

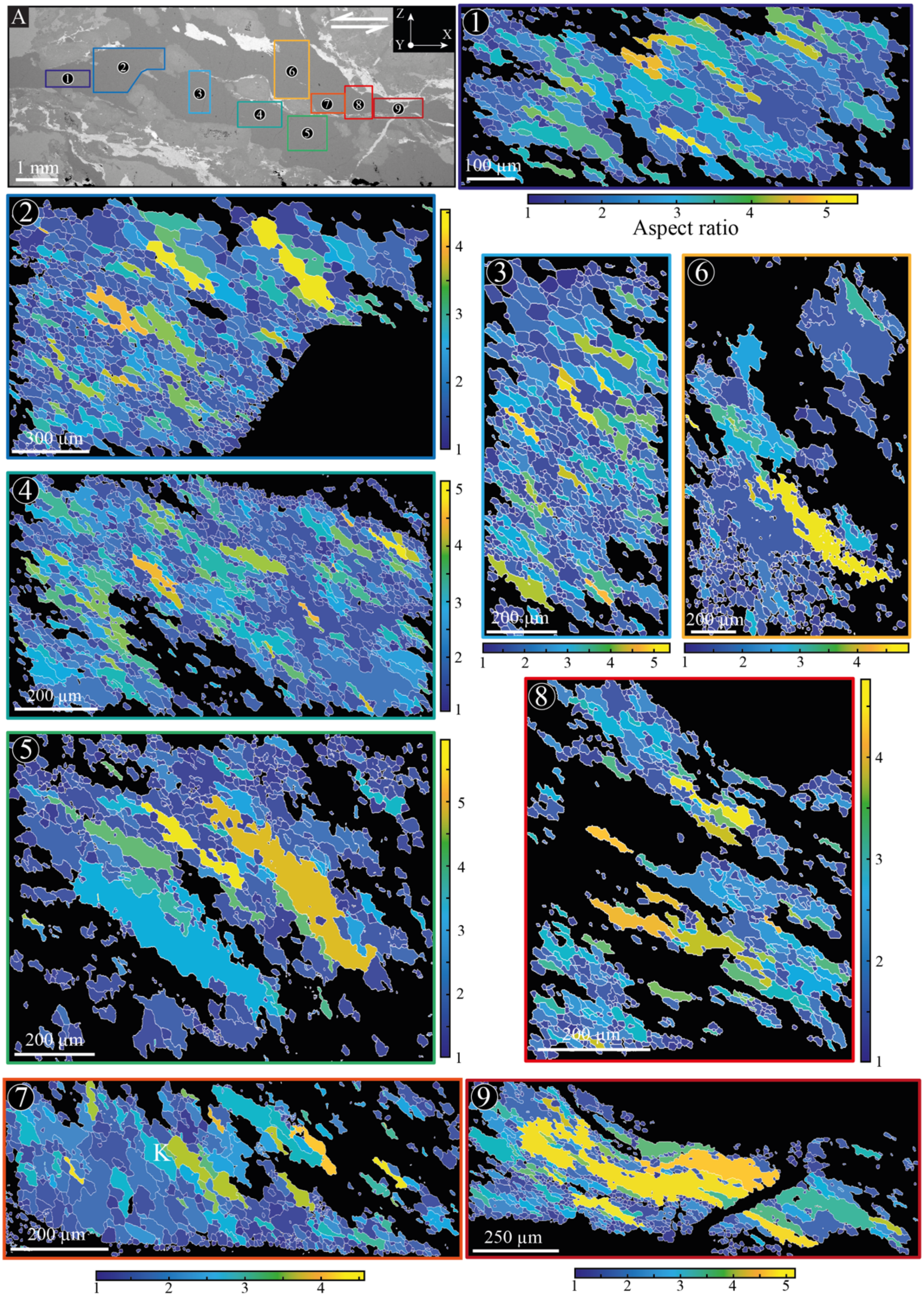

Supplementary figure 5 | EBSD maps of quartz aggregates located in figure 8 of the manuscript. **A**) Backscattered electron images locating all EBSD maps. **1-9**) EBSD maps where quartz grains are highlighted using their aspect ratio (color bar). Border grains and other phases (black areas) are not shown. X = shear direction; Z = pole to shear plane.
